# Mechanisms for Enhancing Radiosensitivity in Esophageal Cancer

**DOI:** 10.3390/cancers18162610

**Published:** 2026-08-13

**Authors:** Dongli Guo, Jing Jin, Xin Su, Wanyu Yang, Bin Guo, Wenpeng Jiao, Yutong He

**Affiliations:** 1 Cancer Institute, Fourth Hospital of Hebei Medical University, 12 Health Road, Chang’an District, Shijiazhuang 050011, China; 49406491@hebmu.edu.cn (D.G.);; 2 Department of Thoracic Surgery, Fourth Hospital of Hebei Medical University, Shijiazhuang 050011, China; 3 Department of Radiation, Fourth Hospital of Hebei Medical University, Shijiazhuang 050011, China

**Keywords:** esophageal cancer, radiotherapy, radioresistance, radiosensitivity, biomarkers

## Abstract

Esophageal cancer is a common malignancy of the upper gastrointestinal tract that is associated with high incidence and mortality rates. Radiotherapy constitutes a cornerstone therapeutic modality for esophageal cancer. In radiotherapy, ionizing radiation is used to eliminate tumor cells through direct DNA damage and indirect reactive oxygen species (ROS)-mediated effects. However, clinical outcomes are frequently limited by interpatient heterogeneity and intrinsic tumor radioresistance. This review systematically describes the determinants of radiosensitivity in esophageal cancer within the established radiobiological framework of the “6Rs”: DNA damage repair (Repair), which is mediated by γ-H2AX phosphorylation, PARP family enzymes, and nonhomologous end joining (NHEJ) and homologous recombination (HR) pathways; cell cycle redistribution (Redistribution), which is regulated by G1/S and G2/M checkpoint dynamics; tumor repopulation (Repopulation), which is driven by cancer stem cell activity during fractionated treatment; reoxygenation (Reoxygenation), which is modulated through HIF-1α signaling and ROS homeostasis; intrinsic radiosensitivity (Radiosensitivity), which reflects interindividual and histopathological variability; and reactivation of antitumor immune responses (Reactivation), which enhances efficacy by remodeling the tumor immune microenvironment. Furthermore, regulated cell death mechanisms, including ferroptosis, autophagy, and apoptosis, significantly modulate radiotherapeutic responses. Elucidating these interconnected mechanisms provides a robust theoretical foundation for developing targeted interventions, identifying predictive biomarkers, and advancing precision radiotherapy strategies to optimize clinical outcomes for patients with esophageal cancer.

## Simple Summary

RT is a cornerstone treatment for esophageal cancer, yet intrinsic and microenvironment-mediated radioresistance severely impairs therapeutic efficacy. This article systematically summarizes esophageal cancer radioresistance mechanisms under the classic 6R radiobiological framework, covering DNA repair, cell cycle redistribution, tumor repopulation, reoxygenation, intrinsic radiosensitivity, and immune reactivation. Crosstalk among apoptosis, ferroptosis, and autophagy further modulates radiation responses. This review also summarizes current targets for radiosensitization and emerging biomarkers. These findings highlight unresolved shortcomings in adenocarcinoma-related evidence and insufficient clinical validation, offering promising directions for subsequent translational exploration.

## 1. Introduction

Esophageal cancer is a common malignancy of the upper gastrointestinal tract and is classified into two primary pathological types, esophageal adenocarcinoma (EAC) and esophageal squamous cell carcinoma (ESCC), with the latter being more common. Esophageal cancer severely affects human health and survival, and its incidence rate is increasing. In 2022, 511,000 new cases of esophageal cancer were reported worldwide along with approximately 445,000 deaths. Moreover, the age-standardized incidence rate (ASIR) and age-standardized mortality rate (ASMR) were 5.00/100,000 and 4.30/100,000, ranking 7th and 6th globally for cancer incidence and mortality, respectively. The incidence and mortality rates in males (9.3/100,000 and 8.2/100,000, respectively) are 2 to 3 times higher than those reported in females (3.6/100,000 and 3.2/100,000, respectively) [1]. The incidence of esophageal cancer in China is high, with ESCC being the predominant subtype, accounting for 85% of the total cases. Currently, surgical resection is the most effective treatment for esophageal cancer. However, many patients cannot undergo surgery because of advanced age, late-stage disease, or tumor location of esophageal cancer particularly T4-stage tumors and cervical esophageal tumors, for which surgical resection is challenging. Consequently, radiotherapy (RT) has become an important treatment option for these patients. Nevertheless, the risk of recurrence remains substantial. Although an increase in the total RT dose or dose per fraction may improve local tumor control, it also increases the risk of toxicity to normal tissues. Therefore, achieving a balance between better therapeutic outcomes and reduced radiation-induced damage to normal tissues needs to be further explored. Specifically, optimizing fractionation schedules by exploiting the differences between early-responding and late-responding tissues may improve local tumor control while minimizing adverse effects on normal tissues. Consequently, RT is often the preferred treatment option for these patients. However, owing to individual differences in treatment response result in radioresistance in some patients, limiting the effectiveness of RT. Consequently, the 5-year survival rate after radical RT is only 30% [2]. Therefore, strategies to reduce the degree of radioresistance in these patients are urgently needed to improve treatment outcomes.

The cellular response to radiation is complex and dynamic and involves various cellular and molecular mechanisms [3]. By using ionizing radiation, RT directly affects the DNA of esophageal cancer cells, thus disrupting molecular structures and causing DNA single- and double-strand breaks (SSBs and DSBs, respectively), which compromise genomic integrity. This phenomenon is one of the key mechanisms through which radiation kills esophageal cancer cells. Additionally, ionizing radiation can generate oxygen free radicals and reactive nitrogen species via water decomposition, which further induces DNA DSBs, damages organelle function, and causes lipid peroxidation, thereby indirectly leading to cell death [4]. Studies focusing on radioresistance in tumor cells have identified several contributing factors. For example, long noncoding RNAs (lncRNAs) can influence the radiosensitivity or resistance of ESCC cells [5]. Other contributing factors include cancer stem cells (CSCs), DNA damage repair capacity, the ability to scavenge reactive oxygen species (ROS), epithelial–mesenchymal transition (EMT), and the abnormal regulation of programmed cell death [6]. The ultimate mechanisms of radioresistance summarized above are generally consistent with the traditional 6R principles, which include repair, redistribution, repopulation, reoxygenation, radiosensitivity, and reactivation of the immune response [7,8]. This review summarizes and discusses the research directions related to traditional 6R principles in the context of esophageal cancer RT. The aim of this study was to elucidate the mechanisms that influence radioresistance in esophageal cancer patients, thereby providing a foundation for the development of improved therapeutic strategies.

## 2. Materials and Methods

The literature for this review was retrieved from PubMed (https://pubmed.ncbi.nlm.nih.gov/ accessed on 11 August 2026), with the cutoff date of 30 April 2026. The search terms included esophageal cancer, ESCC, EAC, RT, radioresistance and radiosensitization. Eligible studies primarily involved in vitro cell experiments and diverse clinical investigations of esophageal cancer patients. Case reports, retracted articles, and literature irrelevant to esophageal cancer RT were excluded. All the retrieved records underwent two rounds of screening: an initial screening of the titles and abstracts, followed by a second screening of the full text. This review has inherent limitations. Most mechanistic studies to date have focused on ESCC, and most radiosensitizing targets have not been validated in large clinical trials. In addition, some regulatory mechanisms may be omitted because of the different experimental protocols used among different studies.

## 3. Repair: The Capacity to Repair Radiation-Induced DNA Damage

Repair here refers to the capacity of cells to repair radiation-induced DNA damage. RT-induced DNA SSBs and DSBs trigger the DNA damage response (DDR). Here, we elaborate on the key mechanisms of DNA damage in esophageal cancer.

Research on esophageal cancer have reported that DNA damage repair-related proteins primarily include γ-H2AX, the PARP (poly ADP-ribose polymerase) family, Ku70/80, DNA-PKcs, RAD51, the MRN complex, ATM/ATR, the XRCC family, and MDC1, among others. γ-H2AX is typically used to assess the DNA repair capacity of esophageal cancer cells [9,10,11]. Nucleosomes (the basic structural units of chromatin) consist of histones and DNA. Among the five histone types, H2A is a core histone, and H2AX is a variant of H2A that replaces traditional H2A in nucleosomes. When DNA is damaged, the Ser139 site of histone H2AX is rapidly phosphorylated, resulting in the formation of the γ-H2AX protein. Therefore, γ-H2AX is usually used as a marker of DNA DSBs [12]. The MDC1 protein plays a critical scaffolding role in DNA damage repair and directly recognizes γ-H2AX [13]. Moreover, H2AX phosphorylation is regulated primarily by ATM, ATR and DNA-PKcs. Under normal conditions, ATM exists as an inactive homodimer. When DSBs occur, ATM undergoes autophosphorylation to form a monomeric structure; become activated; and in conjunction with the MRE11-Rad50-NBS1 (MRN) complex, recruited to the site of damage. At the damaged site, it directly phosphorylates the C-terminal serine residue (S139) of H2AX, thereby leading to γ-H2AX formation [14]. When ATM is deficient or pharmacologically inhibited, DNA-PKcs can compensate to mediate H2AX phosphorylation [15]. In addition, ATM phosphorylates several substrates, including CHK2, BRCA1, 53BP1, and p53, thereby participating in DNA repair processes. SOX17 can transcriptionally repress DNA repair- and DDR-related genes, such as BRCA1, BRCA2, and RAD51, thereby increasing the sensitivity of esophageal cancer cells to chemoradiotherapy [16]. Additionally, p53 translation is regulated by the ROBO1/G3BP2/eIF3A axis [17]. ATR responds primarily to replication stress or regions of single-stranded DNA (ssDNA). When replication forks stall or when the DNA end resection process generates ssDNA, ATR is activated and phosphorylates H2AX through the TOPBP1 and 9-1-1 complex (RAD9-HUS1-RAD1) signaling axis, which specifically maintains the γ-H2AX signal under prolonged replication stress [18]. During the early stages of DNA SSBs or DSBs, PARP1, a member of the PARP family, directly recognizes and binds to DNA damage sites through its zinc finger domain. PARP1 then catalyzes the poly(ADP-ribosyl)ation (PARylation) of both itself and its substrate proteins, thus forming PAR chains that initiate early damage signaling and chromatin remodeling [19,20]. Recent research on esophageal cancer has demonstrated that LNCAROD acts as a scaffold, thereby inhibiting PARP1 degradation via the ubiquitin–proteasome pathway. Moreover, it enhances the protein–protein interaction between PARP1 and NPM1, thereby promoting efficient DNA repair and maintaining genome stability [21]. Additionally, AEG-1 recruits the deubiquitinase USP10 to remove the K48-linked polyubiquitin chain from Lys425 of PARP1, thereby preventing its proteasomal degradation [22]. When DNA DSBs occur, cells activate either nonhomologous end joining (NHEJ) repair or homologous recombination repair (HRR) mechanisms depending on specific cell cycle stage (NHEJ is preferred in G1, whereas HR is favored in S/G2) and the status of DNA ends. Furthermore, the core protein components and functions of these two repair pathways significantly differ.

The core factors involved in the NHEJ repair pathway include Ku70/Ku80 and DNA-PKcs [23]. Ku70/80 initially recognizes and binds to the broken ends of DNA strands, subsequently recruiting DNA-PKcs to form a functional DNA-PK holoenzyme. This recruitment directly activates the kinase activity of DNA-PKcs and triggers its autophosphorylation, which further leads to the recruitment and activation of other essential NHEJ factors, such as DNA ligase IV and XRCC4, ultimately resulting in the assembly of a complete repair complex [24]. Studies have demonstrated that the stability of the Ku70/Ku80 complex in esophageal cancer patients is regulated by VAV2. The SH3 domain at the C-terminus of VAV2 binds to Ku70, thereby mediating NHEJ repair [25]. Additionally, UHRF1 may regulate Ku70 protein expression [26]. Moreover, thymine DNA glycosylase can activate the transcriptional coactivator with the PDZ binding domain (TAZ) by demethylating DNA, which upregulates the expression of NHEJ-related genes such as Ku70 and Ku80, thereby accelerating DNA DSB repair [27]. Moreover, human positive cofactor 4 (PC4) promotes NHEJ repair by increasing XLF expression [28].

The core proteins involved in the HRR pathway primarily include RAD51 and the MRN complex. The main catalytic function of RAD51 is to promote homologous sequence search and strand exchange operations between ssDNA and double-stranded DNA (dsDNA), thereby forming a D-loop structure. Specifically, RAD51 binds to ssDNA to form a filamentous structure known as the RAD51-ssDNA filament, which scans the entire dsDNA of interest to search for homologous sequences. It subsequently promotes strand invasion/exchange to complete DSB repair. Additionally, RAD51 plays a crucial role in replication fork protection and reversal, thereby maintaining genomic stability [29]. DNA polymerase iota (POLI) also promotes HRR capabilities by stabilizing the RAD51 protein, thus contributing to radioresistance. POLI downregulation prolongs the persistence of γ-H2AX foci and inhibits HRR [30]. Berberine directly downregulates RAD51 expression, whereas valproic acid reduces RAD51 levels via histone acetylation (which relaxes the chromatin structure); both these effects increase the number of radiation-induced DSBs [31,32]. The MRN complex is another key protein involved in HRR. It acts as both a “sensor” and “processor” of DNA damage and plays critical roles in DNA recognition, signaling, and repair [33]. Additionally, through its structural properties, the MRN complex can directly bind to DSB sites. The nuclease activity of MRE11 is responsible for the resection of short DNA ends, thus providing ssDNA templates for subsequent HRR steps [34]. The ATPase activity of RAD50 also regulates the DNA binding and dissociation activities of the complex through conformational changes, thereby facilitating the stabilization and ligation of DNA strand break ends [35]. The activation of ATM kinase is also regulated by the MRN complex. This activation triggers cell cycle checkpoint arrest to allow time for repair and the amplification of DNA damage signals through the phosphorylation of downstream target proteins, such as CHK2 [33]. Furthermore, the interaction between MRN and DNA-PK balances the repair pathway selection process. For example, when MRN activity is suppressed (such as through the MIN protein), cells are more likely to use error-prone NHEJ than to employ the precise HRR pathway, thus leading to genomic instability [36].

**Figure 1:**
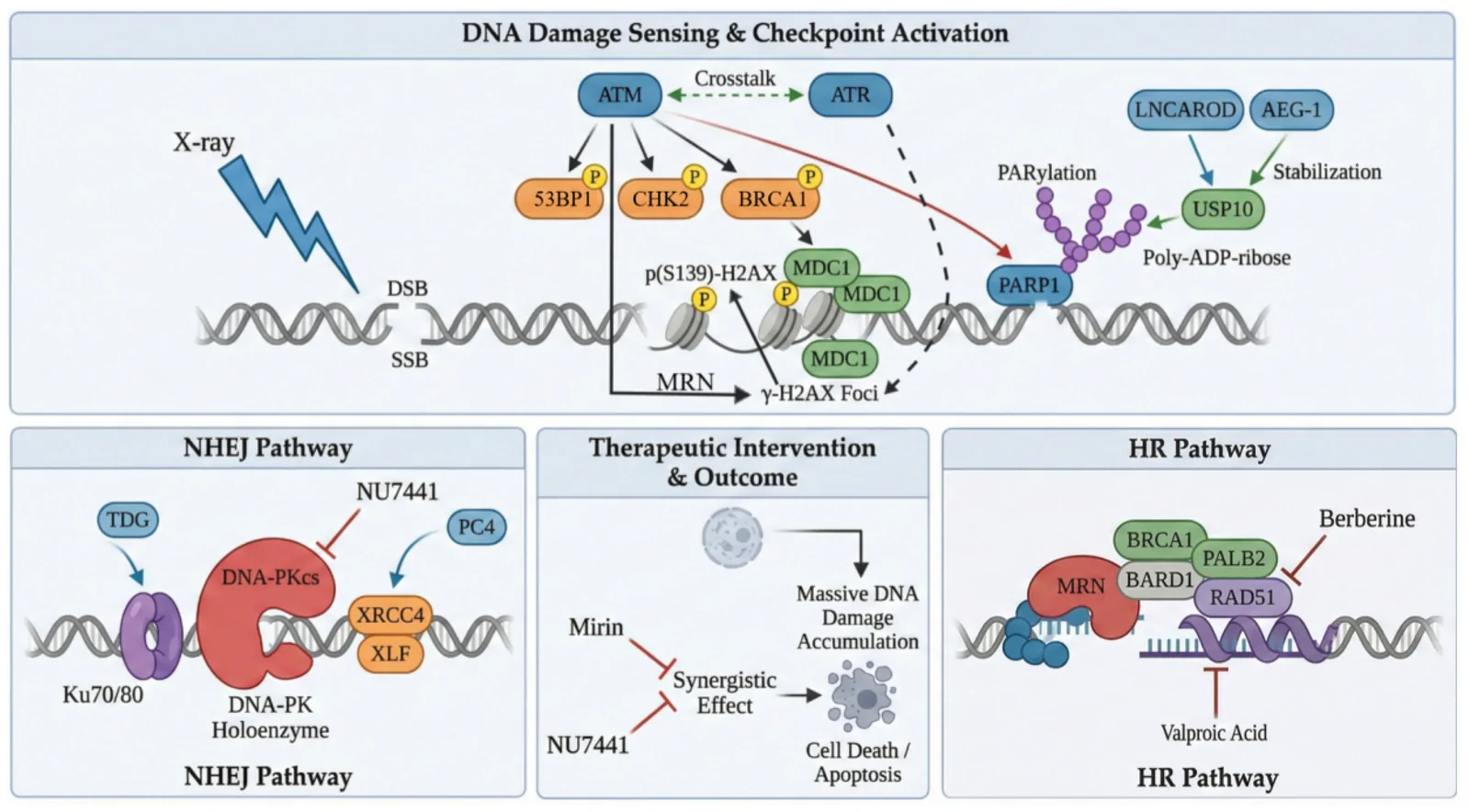
Partial mechanistic diagram of DNA damage repair and radiosensitivity in esophageal cancer cells (note: The pathways shown were compiled from independent studies and do not constitute a single experimentally validated signaling network.).

Additionally, esophageal cancer studies have investigated DNA damage repair, but the mechanisms underlying DNA damage regulation are poorly defined. Research on DNA damage repair signaling pathways in esophageal cancer patients has revealed the following: (1) GPR37 promotes the ubiquitination and degradation of ATP1A1, inhibits the activation of the Akt/mTOR pathway and reduces the DNA repair capacity [37]; (2) ROBO1 disrupts p53 translation by degrading eIF3A and activates the mTOR pathway, thus increasing DNA repair-related gene expression and radioresistance [17]; (3) CYH33, a PI3Kα inhibitor, increases the DNA damage level promotes G2/M phase arrest by blocking radiation-induced Akt/FOXO1 phosphorylation [38]; (4) HMGB1, when activated by Wnt signaling, increases radioresistance by regulating DNA damage checkpoints and DDRs [39]; (5) the METTL3/IGF2BP3/circCREBBP/MYC axis influences MYC expression via RNA m6A modification, thus serving as a potential target for increasing radiosensitivity [40]; (6) under hypoxic conditions, KDM3A/KDM6B overexpression increases cell survival and migration rates, reduces DNA damage through modulation by the MRN complex, and promotes radioresistance [41]; (7) cancer-associated fibroblasts (CAFs) promote the tumor cell DDR by activating the PDGFb/PDGFRb/FOXO1/DNM3OS axis, thereby leading to radioresistance [42]; (8) and the appropriate FLT3 ligand activates the Flt-3 receptor and the downstream PI3K/Akt/BAD signaling pathway, thus increasing radioresistance in ESCC cells [43]. Additionally, other signaling pathways are summarized in Table 1. Several mechanisms that affect the radiosensitivity of esophageal cancer cells from the perspective of DNA damage repair are summarized in Figure 1.

The radiosensitivity of esophageal cancer cells is highly dependent on the dynamic balance of the DNA damage repair capacity. This subsection elaborates on the core mechanisms of DNA damage repair in esophageal cancer, focusing on key DDR-associated proteins (γ-H2AX, PARP1, RAD51, and the MRN complex) and the two major repair pathways (NHEJ and HR). From a translational medicine perspective, PARP inhibitors represent the most feasible candidate drugs currently available. The combined use of inhibitors targeting HR (such as mirin) and NHEJ (such as NU7441) significantly increases the number of DSBs [44]. Targeting HRR/NHEJ repair pathways (such as by inhibiting POLI, RAD51, and PC4) [45], regulating cell cycle checkpoints (such as NS1-BP and 14-3-3σ), modulating key signaling pathways (such as the Akt/mTOR, Wnt, and Hippo pathways), and the use of drugs (such as TH-302) [46], VPA, and PI3Kα inhibitors [38] can effectively decrease the repair capacity of tumor cells, thereby increasing the effectiveness of RT. Transcription factors, such as SOX17 and KDM3A, epigenetic regulators, lncRNAs, and stroma-related targets are applicable only to basic mechanistic investigations. Further in vivo experiments and clinical validation are needed prior to advancing research on drug translation.

**Table 1:** Summary of preclinical studies on DNA damage regulators affecting radiosensitivity in esophageal cancer cells.

Molecule	Effect	Target (s)	Reference (s)
HMGB1	PI3K/Akt/FOXO3A signaling pathway	γ-H2AX	[47]
HMGB1	PI3K/Akt/ATM	ATM	[48,49,50]
USP14	USP14/YAP1	γ-H2AX	[51]
NEDD4L	NEDD4L/KLF5	γ-H2AX	[52]
lncRNA AFAP1-AS1	AFAP1-AS1	ATM/ATR	[53]
RAD18	/	DNA-PKcs	[54]
HNF1A	PI3K/Akt	γ-H2AX	[55]
GPR37	GPR37/ATP1A1/Akt/mTOR	γ-H2AX	[37]
RFC4	p53/p21	γ-H2AX/RAD51	[56]
IGF2BP3	METTL3/IGF2BP3/circCREBBP/MYC	γ-H2AX	[40]

(Note: Effect, key proteins in this process; Target: ultimate target protein).

## 4. Redistribution: Cells in Mitosis Are Mostly Sensitive to Radiation, Whereas Cells in the S Phase Are Mostly Resistant. With Multiple Doses, Cells Progress Through a Phase of the Cell Cycle in Which They Are Especially Sensitive to Treatment, Increasing the Overall Therapeutic Effect

The observed differences in radiosensitivity across the stages of the cell cycle are determined primarily by their DNA repair capacity, checkpoint regulation scheme, and chromosome dynamics. The cell cycle is divided into four main phases: G1, S, G2, and M. Cells exhibit significant variations in their radiosensitivity at different cell cycle stages. Typically, cells are most sensitive to radiation in the G2/M phase, followed by the G1 phase, with late S phase being the least sensitive to radiation [57]. The goal of fractionated RT is to use multiple doses of radiation to redistribute cells from a less radiation-sensitive phase of the cell cycle (such as the S phase) to a more sensitive phase (such as G2/M) during subsequent exposures, thereby increasing cytotoxicity. Furthermore, DNA damage repair mechanisms involved in esophageal cancer RT are tightly regulated by the cell cycle. Specifically, HR is more active during the S and G2 phases, whereas nonhomologous end joining is more crucial during the G1 and early S phases. The high radiosensitivity of mitotic cells is a result of their highly condensed chromosomes. During this phase, the HRR pathway is suppressed, which prevents the efficient repair of radiation-induced DNA DSBs and leads to chromosomal misalignment and spindle abnormalities [58,59]. This phase-specific preference for distinct DSB repair pathways has been consistently validated across multiple preclinical models and constitutes a widely accepted mechanistic framework. Additionally, DNA damage repair is closely associated with the cell cycle, and cell cycle checkpoints play critical roles in maintaining genetic integrity. Radiation-induced DNA damage activates cell cycle checkpoints, which temporarily halts cell cycle progression to the next phase and allows time for repair. While the core functions of these checkpoints are well characterized, the mechanisms enabling checkpoint evasion in esophageal cancer remain incompletely defined.

Key proteins such as ATM-CHK2, ATR-CHK1, p53, p21, and CDK2/4/6 are involved in the G1 phase. ATM governs DSB-triggered G1 arrest by phosphorylating CHK2. However, ATR transmits signals primarily through CHK1 to resolve replication stress during the S/G2 phase; nevertheless, extensive functional crosstalk occurs between these two cascades under pathological conditions or in the case of deficiency [60]. Study-specific observation in esophageal cancer have demonstrated that NS1-BP can inhibit the ATM/CHK1 signaling pathway, thereby increasing the degree of RT-induced DNA damage [61]. The ATM–CHK1 cascade is highly conserved and firmly established, yet evidence for NS1-BP is restricted to preclinical investigations, and prospective clinical validation is lacking. MDM2 binds to p53, thus negatively regulating the p53 protein [62]. Moreover, p53 activates p21, which is a CDK inhibitor that binds to and inhibits the cyclin-CDK2 complex, thereby preventing the cell from entering the S phase and resulting in G1 phase arrest. RFC4 enhances the DNA repair process by inhibiting the p53/p21 signaling pathway, thus conferring resistance to RT in patients with esophageal cancer [56]. The p53–p21 axis is clearly clinically relevant, whereas the translational significance of RFC4 remains speculative owing to the lack of larger scale clinical data. Additionally, METTL14 promotes the maturation of miR-99a-5p via m6A modification. miR-99a-5p targets the 3’UTR of TRIB2, thus inhibiting its expression.; moreover, TRIB2 degrades METTL14 via COP1-dependent ubiquitination and activates HDAC2, thus leading to the deacetylation and transcriptional repression of p21 through the Akt/mTOR/S6K1 pathway, which increases resistance to RT in esophageal cancer patients [63]. This bidirectional regulatory circuit is an attractive preliminary finding, although the supporting evidence originates solely from basic experimental research, and further verification is needed. CDK4/6 also plays crucial roles in the G1 phase, mainly by binding to cyclin D, which activates CDK4/6. Subsequently, the Rb protein becomes phosphorylated, relieving Rb-mediated inhibition of E2F and driving the cell toward the S phase. Baicalein increases the radiosensitivity of esophageal cancer cells by inhibiting cyclin D1/CDK4 activity, thereby inhibiting the transition from the G0/G1 phase to S phase [64]. CDK4/6 stands out as among the most promising targets for clinical translation, whereas natural compounds such as baicalein remain in preclinical testing with limited human data. Additionally, the YAP1-CDK6 positive feedback loop mediates radioresistance by upregulating CDK6 expression and increasing CSC characteristics. The combined use of YAP1 inhibitors (such as CA3) and CDK4/6 inhibitors (such as LEE011) can synergistically weaken this pathway and increase therapeutic efficacy [65,66]. Despite promising preclinical results, uncertainties persist regarding the optimal dosing schedules and toxicological risks of dual YAP1/CDK4/6 inhibition in clinical settings.

Key proteins such as BRCA1, NBS1, ATM, p21, and CDK2 are involved in the S phase. The BRCA1-BARD1 complex recognizes DNA damage sites [67]. In a study on esophageal cancer, NRAGE was shown to stabilize RNF8 and BARD1, thereby promoting DNA DSB repair. Moreover, its downregulation leads to G2/M phase arrest and increased γ-H2AX expression, thus weakening DNA repair capabilities [68]. BRCA1 and NBS1 are well-established guardians of genome stability, but the functional role of NRAGE remains unclear. The cyclin A-CDK2 complex initiates DNA replication by phosphorylating multiple key proteins. When cells respond to DNA damage, p21 upregulation inhibits cyclin A-CDK2, thus helping the cell pause DNA replication and enact repairs, which reduces the risk of accumulating mutations [69]. This regulatory paradigm has been broadly reproduced across tumor models and constitutes a consensus mechanism.

Key proteins such as p53, p21, and CHK2 are involved in the G2 phase. Among them, p53 primarily ensures that damaged DNA does not enter mitosis. Additionally, p21 helps maintain the G2/M checkpoint. CHK2 stabilizes p53 via phosphorylation and prevents CDK1 activation by phosphorylating Cdc25A/C, thus resulting in G2 phase arrest [70,71]. The core signaling network governing the G2/M checkpoint is well documented, with relatively little disagreement among researchers. FOXK1 increases Cdc25A and CDK4 transcription by directly binding to their promoters [72]. Furthermore, LINC00473 reduces the radiosensitivity of esophageal cancer cells by modulating the miR-497-5p/Cdc25A axis [73]. Exosomal miR-339-5p can also target and regulate Cdc25A [74]. All of these regulatory axes have been described only in preclinical studies, leaving substantial gaps in translational knowledge. Additionally, ZNF468 increases radioresistance by upregulating the expression of the Aurora A protein, which reduces p53 expression, thereby weakening G2/M phase arrest and apoptosis [75]. Although mechanistically intriguing, available evidence for ZNF468 is limited, and its prospects for clinical application remain unclear. Mitotic entry is primarily controlled by both p53 and 14-3-3σ proteins at the G2/M checkpoint. Although p53 predominantly affects phases G1 and G2, it also indirectly blocks the G2-to-M transition by activating 14-3-3σ transcription. Studies have demonstrated that in esophageal cancer patients, 14-3-3σ increases NHEJ efficiency by promoting the recruitment of PARP1 and DNA-PKcs; it also induces CHK2 expression, thus triggering G2/M cell cycle arrest that results in sufficient time for DNA lesion repair [76]. These findings are partially supported by patient-derived samples, yet validation in larger independent cohorts is still needed. Cdc2 kinase activity can be maintained in cancer cells by activating the CHK2/Cdc25C/Cdc2 signaling pathway or overexpressing Cdc25B, thereby abrogating G2/M arrest and promoting apoptosis [77,78,79]. Cdc25B represents a candidate predictive biomarker, but consistent multicohort validation is needed before its clinical implementation. The G1/S and G2/M checkpoints are the two most crucial checkpoints in the cell cycle and occur at the early and late stages of the cell cycle, respectively. Moreover, p53 plays a core role in regulating both checkpoints, with more direct and significant control being exerted at the G1/S checkpoint. This principle represents fundamental, largely undisputed finding in cell cycle biology. As previously noted, the cell cycle and DNA repair are tightly coordinated. When DNA damage is sensed, proteins such as the MRN complex and BRCA activate ATM and other proteins [80], which then phosphorylate downstream targets such as CHK2 to coordinate cell cycle arrest and repair responses [57]. The integrated cascade linking damage sensing, checkpoint activation and DNA repair has been systematically characterized. After a single exposure to radiation, tumor cells temporarily arrest at checkpoints such as G1/S and G2/M to repair DNA damage. At the signaling pathway level, the Hippo–YAP1 axis increases radiosensitivity by suppressing the transcriptional activity of KIF3B via YY1, thus leading to the phosphorylation and subsequent reduced nuclear expression of YAP1 [81]. As a downstream effector, targeting the Wnt/β-catenin/CCNG1 axis can also increase resistance to RT [82]. Both pathways represent promising avenues for future investigation, although current mechanistic data are confined to preclinical systems. In terms of epigenetic mechanisms, METTL14 promotes miR-99a-5p maturation via m6A modification, inhibits TRIB2 expression, and activates the Akt/mTOR/S6K1 pathway, ultimately increasing stem cell properties and radioresistance through p21 deacetylation [63]. Epigenetic modulation of cell cycle control is a rapidly evolving field; however, existing evidence remains fragmented, and complete regulatory networks have yet to be established. Additionally, miR-29, miR-497-5p, miR-4443, and miR-200c increase radiosensitivity by regulating key proteins such as cyclin B1, Cdc2, and p21 [73,83,84,85]. Numerous miRNAs that modulate radiosensitivity have been identified, yet major translational obstacles, including poor stability and inefficient in vivo delivery, remain unresolved.

**Figure 2:**
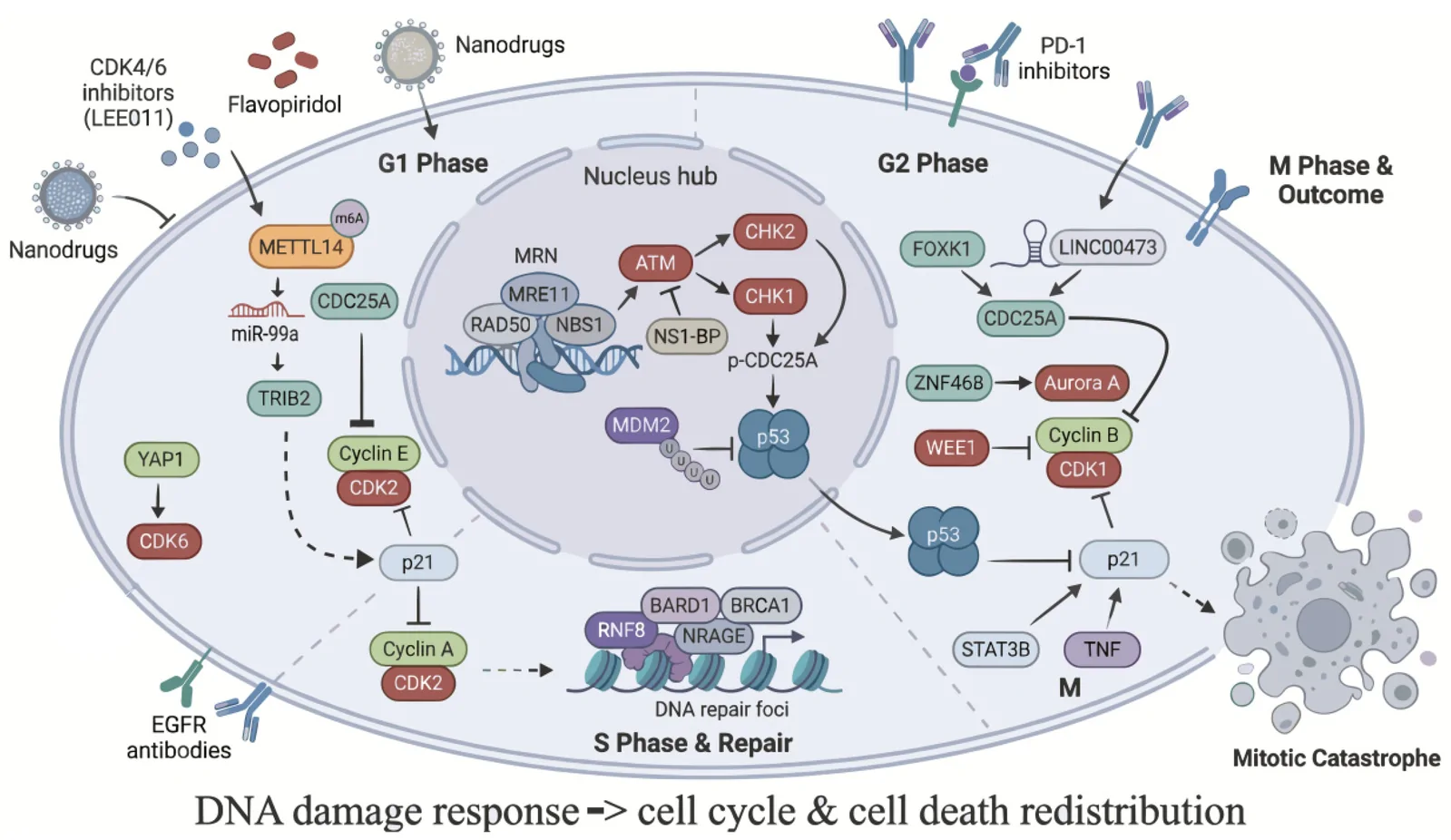
Partial mechanistic diagram of the cell cycle and radiosensitivity in esophageal cancer cells (note: The pathways shown were compiled from independent studies and do not constitute a single experimentally validated signaling network.).

Radiosensitivity across cell cycle phases is governed by phase-specific DNA repair capacity, checkpoint regulation, and chromatin dynamics. Fractionated RT exploits cell cycle redistribution to increase DNA damage in radiosensitive phases (G2/M). Therapeutic targeting of the cell cycle in esophageal cancer represents a promising approach for increasing tumor radiosensitivity during RT. Moving forward, quantitative clinical analyses are needed to define the actual magnitude of benefit achievable through cell cycle redistribution strategies. Numerous studies have recently investigated the roles of p53, p21, and ATM in increasing the radiosensitivity of esophageal cancer cells. Studies have demonstrated that cetuximab reverses resistance to radiation by blocking EGFR expression [86], whereas nimotuzumab increases radiosensitivity by upregulating IGFBP-3 expression [87]. Additionally, the Smad3-P27/p21-cyclin E1/CDK2 pathway mediates the regulation of radiosensitivity by IGFBP-3 [88]. Clinical data supports cetuximab and nimotuzumab as relatively feasible translational candidates, whereas additional clinical evidence is necessary to confirm the functional role of IGFBP-3. Various traditional Chinese medicine extracts have also been widely investigated. For example, baicalin inhibits G1 phase progression by downregulating cyclin D1/CDK4 [64], and astaxanthin increases radiosensitivity by inducing G2/M arrest and apoptosis [89]. Moreover, the flavonoid pyridine inhibits CDK1/2 and increases radiation-induced apoptosis [90]. However, these natural agents have been assessed only in preclinical models, and their ambiguous molecular targets and standardization challenges substantially hinder their clinical translation. Owing to its low toxicity and efficient delivery capabilities, the multifunctional biomimetic drug CMEC-Dbait has emerged as a potential radiosensitizer [91]. While biomimetic delivery platforms are conceptually attractive, CMEC-Dbait itself remains at an early experimental stage. In terms of immunotherapy, PD-1 inhibitors promote G2/M arrest and DNA damage through an IFNγ-dependent pathway [92]. By contrast, STAT3β induces programmed necrosis and inhibits cell cycle progression by activating the TNF signaling pathway [93]. Crosstalk between immune checkpoint signaling and cell cycle control constitutes an emerging frontier, although many unresolved mechanistic questions remain. Furthermore, cell cycle-related biomarkers are becoming increasingly recognized for their ability to predict RT responses; specifically, high COX2 expression is associated with radiochemotherapy resistance [94]. Cdc25B serves as a predictive factor for the p53-independent G2/M checkpoint [78]. Furthermore, high CDCA2 expression is linked to cell cycle changes and resistance to radiation [95], and biomarkers such as lncRpph1 [96], miR-29, miR-497-5p, miR-4443, and miR-200c [73,83,84,85] also increase radiosensitivity. The related mechanistic diagrams are shown in Figure 2. Most candidate biomarkers await validation in large, independent clinical cohorts. Among all the molecular targets discussed, CDK4/6, ATM/CHK1 and the p53 axis have been prioritized for subsequent translational research.

## 5. Repopulation: Cells Proliferate Between Dose Fractions and the Intrinsic and Individual Radiosensitivity of the Target Tumor Affects the Response to RT

During fractionated RT, tumor cells proliferate after each round of treatment because of the presence of residual tumor cells. The principal rationale for conventional fractionation is to spare late-responding normal tissues, whereas the risk of tumor repopulation supports minimizing overall treatment time (e.g., accelerated or hyperfractionated schedules). However, the optimal fractionation schedule that ensures a balance among tumor control, the tolerance of normal tissues, and tumor repopulation remains controversial, and no consensus has been reached in clinical practice. Here, we discuss the stem-like characteristics of esophageal cancer cells, such as self-renewal, differentiation potential, and migration and invasion capabilities. These properties allow surviving tumor cells to maintain stemness following radiochemotherapy, driving therapeutic resistance and tumor relapse. Although this mechanistic framework holds broad consensus and promising translational value, clinical trials validating CSC-targeted interventions remain severely limited. Studies have demonstrated that SOCS6 overexpression promotes the radiation-induced ubiquitination of c-Kit, thereby reducing its protein level and weakening the stem cell characteristics of ESCC cells, which increases their radiosensitivity [97]. This regulatory axis (SOCS6/c-Kit) is supported by robust in vitro and xenograft data and is thus a well-validated preclinical mechanism with promising therapeutic potential. Moreover, ΔNp63α can regulate RSK4 transcription, thereby phosphorylating Ser9 of GSK-3β, which activates the Wnt/β-catenin signaling pathway, thus promoting CSC characteristics and radioresistance [98]. The ΔNp63α/RSK4/GSK-3β/Wnt/β-catenin pathway has been mechanistically verified, but its cross-talk with other signaling pathways and tissue-specific effects in esophageal cancer remain poorly defined, indicating a critical knowledge gap. The TRIB3 protein binds to TAZ and inhibits its β-TrCP-mediated ubiquitination and degradation, thereby increasing its stability and activity. This effect further promotes CSC traits, thus increasing the resistance of ESCC cells to RT, which ultimately results in poor patient prognosis [99]. Notably, the TRIB3/TAZ axis has been validated exclusively in preclinical ESCC models, with no clinical correlative data or translational studies conducted to date; this highlights a major limitation for clinical application.

From a translational medicine perspective, among the identified targets, SOCS6/c-Kit holds the most promise for therapeutic development because of its consistent validation in preclinical studies and clear mechanistic link to radiosensitivity. In contrast, further in vivo optimization and clinical verification are needed for ΔNp63α and TRIB3/TAZ before translational research can be initiated. Key controversies remain regarding the relative dominance of these pathways in CSC-mediated radioresistance, and large knowledge gaps exist in the translation of these preclinical findings to EAC and clinical patient cohorts.

## 6. Radiosensitivity: The Response to Radiation Varies According to Intrinsic and Individual Tumor Radiosensitivity

From a classical radiobiological standpoint, inherent cellular radiosensitivity is defined as the fifth “R” governing the tumor response to radiation. Different types of tumor cells exhibit various inherent sensitivities to radiation, and low-dose rate survival curves better reflect the differences between tumors than acute survival curves do. Additionally, individual cell lines display notable disparities in sensitivity to initial radiation-induced lethal damage, which serves as a core driver of differential radiosensitivity independent of postirradiation DNA repair capacity.

When these preclinical radiobiological findings are translated to esophageal cancer subtypes, no direct mechanistic or prospective clinical evidence is currently lacking, which suggests that ESCC is inherently more sensitive to RT than EAC is. Geographic distributions of the two histological subtypes notably differ: ESCC is more common in Asian countries, whereas EAC is more prevalent in Western countries [100]. Available evidence indicates that after neoadjuvant therapy, the pathological complete response (pCR) rate in ESCC patients is 32.4%, which is higher than the 25.2% reported in adenocarcinoma patients. While these clinical response data indirectly imply a better overall radiotherapeutic response among ESCC patients, they do not fully distinguish the independent contribution of inherent cellular radiosensitivity from that of other radiobiological factors, including repopulation, reoxygenation and CSC-mediated radioresistance, representing a critical knowledge gap restricting the precise translational interpretation of clinical outcomes.

Recent clinical statistics do not exclude multiple confounding variables, such as tumor stage at diagnosis, patient baseline comorbidities and institutionally divergent chemoradiotherapy regimens, Thus, we cannot draw a definitive conclusion that EAC cells intrinsically possess stronger radioresistance.

Taken together, these comparative clinical results suggest that compared with EAC patients, ESCC patients achieve a better response to RT to a certain extent, although further stratified basic and clinical research is needed to verify whether this gap occurs because of intrinsic cellular radiosensitivity.

## 7. Reoxygenation: Sensitivity to Radiation Increases in Well-Oxygenated Tissues

In this section, we discuss the effects of different oxygen concentrations on the effectiveness of RT in esophageal cancer cells. Traditionally, reoxygenation refers to a dynamic change in the tumor microenvironment (TME) during fractionated RT. Factors such as reduced metabolic activity, improved blood circulation, tumor shrinkage, and angiogenesis work together to allow tumor cells that were previously hypoxic to regain their oxygen supply, which increases their sensitivity to radiation. Compared with a single high dose of irradiation, fractionated RT results in less severe late toxicity to normal tissues when administered at an equivalent tumor control dose, as smaller fractional doses effectively spare late-responding normal organs.

Research on oxygen levels in esophageal cancer patients has focused primarily on the central role of hypoxia-inducible factor (HIF-1α) and the influence of dynamic ROS balance on RT sensitivity. HIF-1 is a heterodimeric transcription factor consisting of two subunits: HIF-1α (the oxygen sensitive functional subunit) and HIF-1β (the structural subunit). HIF-1α remains stable under hypoxic conditions and is rapidly degraded under normoxia, whereas HIF-1β is stably expressed under different oxygen conditions. This structure allows HIF-1 to sense changes in the oxygen concentration and regulate target gene expression through transcriptional activation [101]. In esophageal cancer research, YAP has been shown to directly regulate the expression of HIF-1α, which functions in conjunction with the hypoxic microenvironment to promote tumor cell survival. YAP and HIF-1α, which together promote tumor growth and the formation of a hypoxic microenvironment, have been shown to interact [102]. USP28 stabilizes the c-Myc protein and promotes HIF-1α accumulation at the transcriptional level, thereby reducing the radiosensitivity of esophageal cancer cells [103]. Baicalein and berberine can target HIF-1α to inhibit its nuclear translocation and protein expression and thereby reverse radioresistance in hypoxic cells [64,104].

Among the above hypoxia-related regulatory axes, the HIF-1α/YAP signaling cascade is associated with the most robust and consistent preclinical data across ESCC and EAC cell lines, patient-derived xenograft models, and transcriptomic analyses of patient cohorts, representing the most well-supported mechanism linking hypoxia to esophageal cancer radioresistance. In contrast, USP28/c-Myc/HIF-1α signaling has been validated in only a limited number of in vitro cell line experiments, and orthotopic tumor model verification and clinical tissue correlation data are lacking, which restricts its immediate translational advancement. In the nonsurgical management of locally advanced ESCC, PD-1 inhibitors facilitate vascular normalization, ameliorate tumor hypoxia, and increase intratumoral oxygen perfusion. These alterations substantially increase tumor radiosensitivity and local disease control, resulting in an improved 2-year local control rate of 81.7% compared with the historical control rate of 71.3% [105,106]. Natural small-molecule inhibitors (e.g., baicalein and berberine) have exhibited radiosensitizing effects in in vitro hypoxic culture systems only, with their poor bioavailability and nonspecific off-target effects in vivo remaining unresolved. These findings indicate that these agents cannot be directly assessed in clinical trials. Conversely, PD-1 immune checkpoint inhibitors stand out as the clinically validated intervention targeting tumor reoxygenation. In phase II clinical trials, combining anti-PD-1 therapy with neoadjuvant chemoradiotherapy improved tumor oxygenation and pCR rates in patients with locally advanced esophageal cancer, forming a feasible translational strategy with a clear clinical evidence base.

Here, we primarily address ROS homeostasis. In terms of the dynamic balance of ROS levels, RT causes water molecules to break down and generate ROS, such as hydroxyl radicals and superoxide anions, which primarily cause tumor cell death by damaging macromolecules such as DNA, lipids, and proteins. When the RT-induced ROS level exceeds the antioxidant capacity of a cell, tumor cell death occurs via various mechanisms, including apoptosis, autophagy, and ferroptosis [107]. Residual tumor cells that remain after RT are often in a hypoxic state. The hypoxic TME limits ROS generation, thus leading to radioresistance [108]. In esophageal cancer patients, Nrf2 activation promotes its nuclear translocation and upregulates the expression of antioxidant genes (such as SLC7A11 and GPX4). These changes trigger a reduction in ROS accumulation and lipid peroxidation, inhibition of ferroptosis, and an increase in radioresistance [109,110]. KLF5 (an activator of Nrf2) increases esophageal cancer radioresistance by inhibiting Keap1 transcription to activate the Nrf2 pathway [111]. Low SNPH expression leads to the redistribution of mitochondria to the cell periphery, thereby reducing radiation-induced oxidative damage and increasing radioresistance [112]. Furthermore, some studies have demonstrated that MnSe2-lipid triggers a Fenton-like reaction through the release of Mn^2+^ ions, thus activating the cGAS-STING pathway, promoting immune cell infiltration, and inhibiting tumor growth [113]. IDH2 knockdown exacerbates radiation-induced ROS overload and mitochondrial dysfunction, thus promoting apoptosis and increasing radiosensitivity [114]. MeJ increases the sensitivity of KY170R cells to RT by inhibiting AKR1C3 activity, reducing the PGF2 production rate, and increasing intracellular ROS levels [115]. Moreover, sulconazole triggers oxidative stress and inhibits glycolysis by downregulating HK expression and inhibiting the activity of PI3K/Akt, MEK/ERK, and STAT3 pathways [116]. The small molecule pyrazinib (P3) significantly inhibits angiogenesis and metabolism, particularly especially oxidative phosphorylation, reduces the secretion of inflammatory factors (such as IL-6 and IL-8), and decreases the ATP production rate [117]. Additionally, studies have demonstrated that p53 mutations increase antioxidant stress responses by upregulating SLC7A11; furthermore, SLC7A11 knockdown can restore radiosensitivity [118].

**Figure 3:**
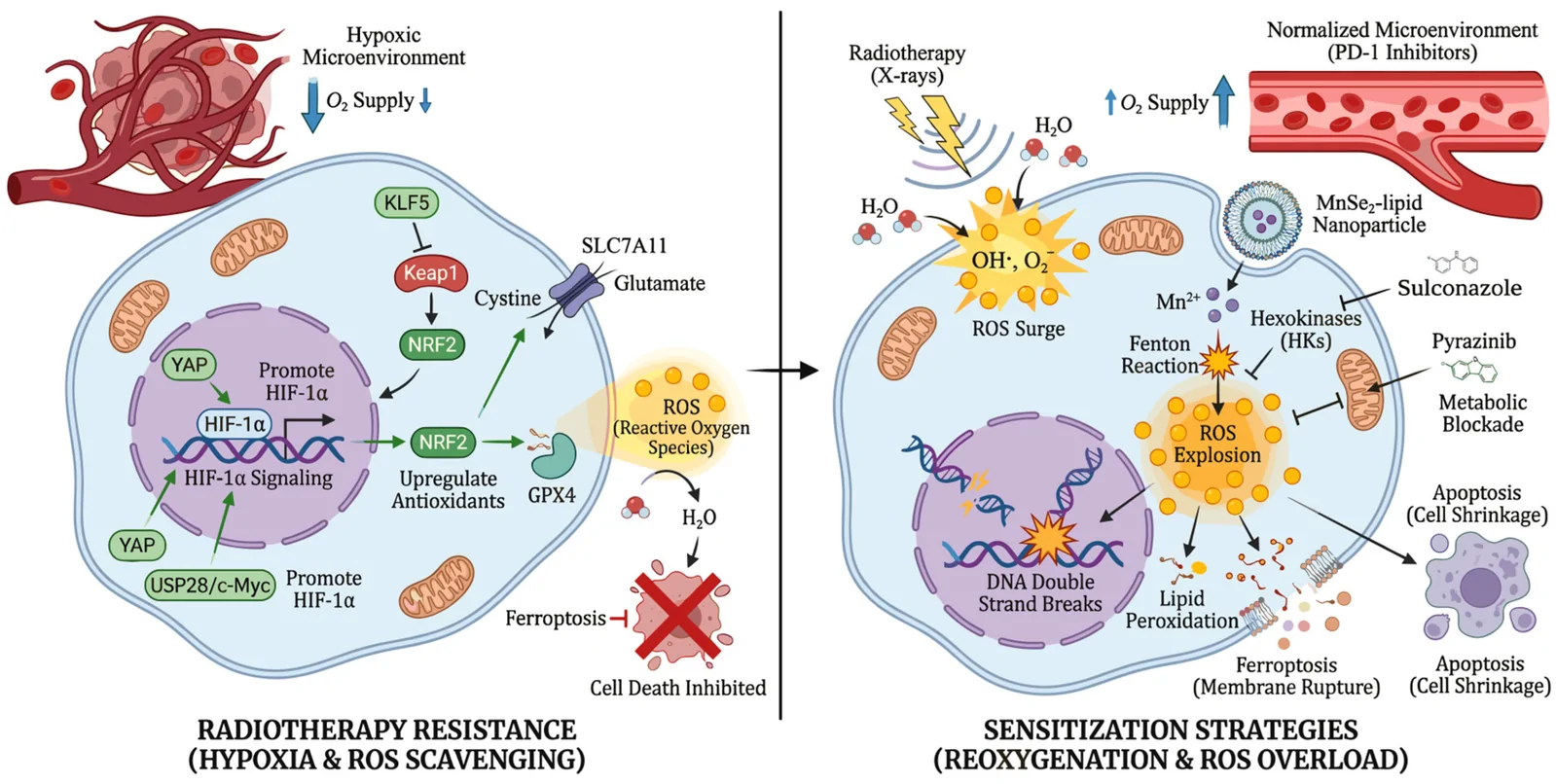
Mechanisms through which reoxygenation and metabolism regulate the radiosensitivity of esophageal cancer cells (note: The pathways shown were compiled from independent studies and do not constitute a single experimentally validated signaling network.).

Overall, the radiosensitivity of esophageal cancer cells strongly depends on the dynamic balance of oxygen levels and ROS in the TME. Hypoxic conditions promote radioresistance through the activation of the HIF-1α/YAP pathway, whereas antioxidant stress pathways (such as the Nrf2 pathway) weaken the effects of RT through the clearance of ROS. Targeting the Nrf2–HIF-1α axis, modulating ROS homeostasis (such as with ML385 and MeJ), improving tumor oxygenation (such as with PD-1 inhibitors), and inducing metabolic reprogramming (such as with sulconazole and pyrazinib) represent promising strategies for overcoming radioresistance. The related mechanistic diagrams are shown in Figure 3.

From a translational medicine perspective, PD-1 inhibitors represent the most clinically feasible strategy: they normalize tumor vasculature to relieve hypoxia, with synergistic benefits demonstrated for esophageal cancer when combined with chemoradiotherapy, supporting their immediate clinical translation. The HIF-1α/YAP and Nrf2/SLC7A11 axes are promising preclinical targets validated across multiple tumor models; specific inhibitors such as ML385 reverse radioresistance, although clinical evidence is still lacking. Natural extracts, metabolic inhibitors and MnSe2-lipid nanomaterials remain preliminary candidates because of their poor in vivo stability and nonspecific toxicity; thus, further preclinical optimization is needed. Major unresolved issues include conflicting reports of SNPH-mediated oxidative stress regulation, unclear functions of USP28 subtypes and the unvalidated predictive value of KLF5. Notably, nearly all relevant studies have relied on ESCC models and lack data on EAC, which has hindered cross-subtype clinical translation.

## 8. Reactivation of the Antitumor Immune Response

Tumors exhibit three developmental stages: immune surveillance, equilibrium, and escape. During the escape phase, tumors suppress immune system functions through various mechanisms, thus creating an immunosuppressive microenvironment [119]. RT not only affects tumor cells via direct cytotoxicity but also remodels the TME, thereby triggering local and systemic immune responses. Reactivation aims to reverse this process, allowing the suppressed immune system to regain its ability to recognize and eliminate tumor cells. Conversely, RT can induce immunogenic cell death (ICD) in tumor cells, thus releasing damage-associated molecular patterns (DAMPs) and tumor-associated antigens that activate the innate immune system and enhance adaptive immune responses [120]. ICD is characterized by the translocation of calreticulin (CRT), release of the high mobility group box 1 (HMGB1) protein, and release of ATP after apoptosis [121]. RT can promote the infiltration of various immune cells (including CD8+ T cells, NK cells, and macrophages) into the TME. However, it can also recruit immunosuppressive cells, such as regulatory T cells (Tregs), myeloid-derived suppressor cells (MDSCs), and M2 macrophages, thereby leading to a dynamic balance between immune suppression and immune activation [122]. Rt-triggered ICD constitutes the most well-verified core mechanism linking radiation and antitumor immunity, with consistent validation reported in in vitro cell models, esophageal xenograft systems and clinical patient tumor samples; this pathway provides a solid theoretical foundation for radiochemotherapy–immunotherapy combination regimens. Nevertheless, the bidirectional effect of RT on immune cell infiltration remains incompletely characterized in clinical cohorts, representing a prominent knowledge gap: few prospective human trials have tracked dynamic changes in CD8+ T cells, Tregs and MDSCs in esophageal tumor tissues across fractionated RT cycles.

Radiation therapy exerts a dual regulatory effect on antitumor immune responses. Although RT fractionation regimens, including fractional dose and total treatment sessions, can trigger robust immune activation, conventional RT frequently yields immunosuppressive effects and impairs systemic immune activation in tumor-bearing hosts. Exosomes secreted by ESCC cells contain miR-143-3p. These exosomes deliver this microRNA to macrophages, where it targets key regulatory factors to promote macrophage polarization toward a tumor-promoting M2-like phenotype (CD206+/Arg-1+), thus forming an immunosuppressive microenvironment [123]. Moreover, tumor-associated macrophages (TAMs) promote radioresistance by upregulating VEGFA and activating its downstream MAPK, BCL-2, and Snail signaling pathways [124]. RT can alter CAF activity and the composition of the extracellular matrix while promoting fibrosis via the differentiation of monocytes into M2 macrophages. These changes in matrix remodeling can affect the physical barrier of tumors and immune cell functions [125,126]. Some studies have suggested that CXCL1 secreted by esophageal cancer cells forms a positive feedback loop with CAF-derived collagen. Collagen increases DNA repair capacity and induces CXCL1 secretion, further activating CAFs through the CXCR2/STAT3 signaling pathway and creating a collagen-rich TME that significantly increases radioresistance [127]. Among stroma-mediated resistance pathways, the miR-143-3p exosome/M2-TAM and CXCL1-CAF-collagen axes display robust reproducibility across multiple preclinical ESCC models, while evidence supporting CAF-mediated fibrosis after irradiation is limited to experiments in single cell lines without EAC subtype validation. Notably, no clinical interventions targeting TAM repolarization or CXCL1/CXCR2 signaling have been validated in esophageal cancer patients, restricting their immediate translational application.

**Figure 4:**
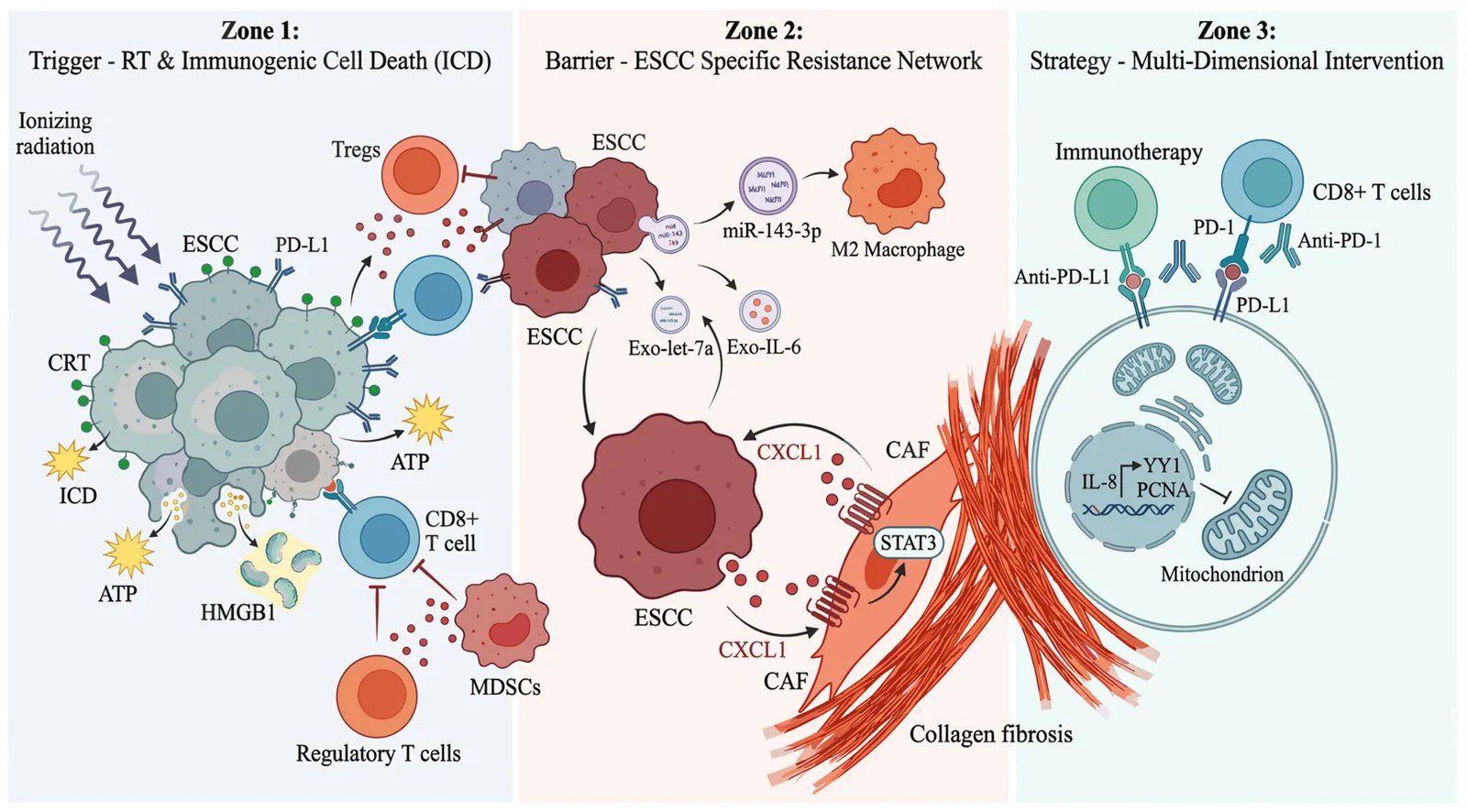
Partial mechanistic diagram of the reactivation of the antitumor immune response and radiosensitivity in esophageal cancer cells (note: The pathways shown were compiled from independent studies and do not constitute a single experimentally validated signaling network.).

RT can induce metabolic changes in tumor cells (such as FABP-dependent lipogenesis), and these metabolic alterations influence other cells in the TME through metabolite exchange [128]. Additionally, immune checkpoints represent promising targets for increasing radiation-induced immune responses. In esophageal cancer patients, radiation can induce the expression of proinflammatory cytokines such as interleukin-1β, tumor necrosis factor α, and type 1 and 2 interferons. Exosomes secreted by sensitive cells containing let-7a can increase the radiosensitivity of resistant cells, whereas those secreted by sensitive cells containing IL-6 can reduce the radiosensitivity of sensitive cells [129]. IL-8 reduces the stability of PCNA mRNA by regulating the expression of miR-27b-5p and increases PCNA gene transcription by upregulating the transcription factor YY1, thus suppressing the effectiveness of RT [130]. Furthermore, RT can upregulate the expression of MHC class I molecules on tumor cells and regulate their peptide repertoire to counteract tumor immune evasion, thereby enabling cytotoxic CD8 T cells to recognize tumor cells. Nevertheless, despite the complexity of tumor immunology and dynamic immunological alterations following RT, targeted strategies have been developed to increase radiosensitivity in the context of esophageal cancer. Studies have demonstrated that RT can modulate the expression of immune checkpoints such as PD-L1 [131]; moreover, in some cases, RT can reduce PD-L1 levels on the surfaces of tumor cells. Given the various metabolites that are generated during cellular metabolic reprogramming, which affect not only the energy metabolism of cancer cells themselves but also the reshaping of the tumor immune microenvironment through alterations in metabolite levels, epigenetic states, and intercellular signaling, we can attribute metabolic reprogramming to the reactivation of antitumor immune responses. The related mechanistic diagrams are shown in Figure 4.

## 9. RT and Cell Death

The efficacy of RT in esophageal cancer patients is closely linked to various programmed cell death mechanisms, such as ferroptosis, autophagy, and apoptosis. These processes regulate radiosensitivity through complex signaling networks, and crosstalk among ferroptosis, autophagy and apoptosis constitutes an undervalued core regulatory logic that jointly determines the fate of irradiated esophageal tumor cells. Here, we generally summarize the current literature related to esophageal cancer. During the regulation of ferroptosis, the EZH2-HIF-1α complex inhibits ACSL4 expression via PRC2-mediated H3K27me3 modifications, thereby weakening ferroptosis and decreasing radiosensitivity [132]. Conversely, ursolic acid increases radiosensitivity by triggering ferroptosis via activation of the p53/SLC7A11/GPX4 signaling pathway [133]. The downregulation of PLK1 expression modulates cancer radiosensitivity by inhibiting the pentose phosphate pathway (PPP), which reduces intracellular NADPH and GSH levels, promotes lipid peroxidation, and ultimately increases the responsiveness to RT [134]. Moreover, ACAT2 knockout positively regulates tumor radiosensitivity by increasing intracellular ROS levels and suppressing GPX4 activity, thereby promoting ferroptosis and potentiating the efficacy of RT [135]. Nrf2 inhibits ferroptosis by upregulating SLC7A11 expression, which contributes to reduced radiosensitivity and the development of tumor radioresistance [136]. Notably, almost all data on ferroptosis regulators are solely derived from preclinical experimental studies, and no targeted ferroptosis agents have entered esophageal cancer clinical trials, resulting in a major translational gap.

In terms of autophagy regulation, studies have demonstrated that radiation can activate the LKB1-AMPK-LC3B pathway, thereby inducing a protective autophagic response that helps cancer cells adapt to radiation-induced damage and decreases radiosensitivity [137]. Additionally, high expression of eEF2K increases autophagic activity, thus promoting the survival of tumor cells and leading to RT resistance; moreover, the inhibition of eEF2K reduces autophagy and increases the effectiveness of RT [138]. Similarly, BTN3A1 interacts with ULK1 and promotes its phosphorylation, thereby initiating autophagy and increasing radioresistance; this effect is positively regulated by HIF-1α signaling [139]. Furthermore, HMGB1 plays a key role in regulating autophagy and RT sensitivity. HMGB1 deficiency leads to reduced autophagic activity, which reverses RT resistance in tumor cells [140]. The Nrf2/CaMKIIα/autophagy signaling axis has also been identified as a critical RT resistance pathway in ESCC cells. Specifically, the overexpression of Nrf2 increases CaMKIIα levels, which promotes Beclin1 activation and the conversion of LC3-I to LC3-II while inhibiting mTOR phosphorylation and p62 expression, thus significantly increasing autophagic activity [141]. Protective autophagy triggered by radiation may serve as a context-dependent mechanism of radioresistance in esophageal cancer, and the Nrf2/CaMKIIα/Beclin1 axis constitutes a critical molecular bridge linking autophagy and ferroptosis: Nrf2 simultaneously blocks ferroptosis via SLC7A11 and promotes cytoprotective autophagy, resulting in dual resistance to RT. The HIF-1α-BTN3A1-ULK1 cascade further connects the hypoxic TME to autophagy activation, revealing multilayered crosstalk between microenvironmental stress and cell death programs. All autophagy-targeted strategies remain confined to preclinical models, with conflicting reports on whether autophagy inhibition uniformly improves the RT response across distinct esophageal tumor subtypes—a prominent unresolved controversy.

In terms of apoptosis mechanisms, POM-1 increases ATP levels by inhibiting CD39, thereby activating the Caspase-9/Caspase-3 pathway to facilitate tumor cell apoptosis and increase radiosensitivity [142]. TRAIL binds to cell surface receptors (DR4/DR5) and recruits the FADD adapter molecule to directly activate caspase-8, which activates caspase-3, thereby increasing esophageal cancer apoptosis and RT sensitivity [143]. Other signaling pathways also increase radiosensitivity in esophageal cancer cells. RG108 induces G2/M phase arrest by regulating the balance between Bcl-2 and Bax [144]. PELI1 inhibits the nonclassical NF-κB pathway via the ubiquitination of NIK, thereby reducing Bcl-XL expression [145]. The knockdown of BMI-1 induces cell cycle arrest via P16/cyclin D2/CDK4 and MCL-1 regulation [146]. Apoptosis represents the canonical cell death modality pursued in clinical chemoradiotherapy. TRAIL/DR4/DR5 extrinsic apoptotic signaling has been validated in patient-derived tumor samples. Anti-apoptotic Bcl-2 family proteins (Bcl-XL and MCL-1) are shared downstream effectors that simultaneously counteract ferroptosis and apoptosis, highlighting the intrinsic crosstalk between these two cell death programs and tumor radiosensitivity. Studies on noncoding RNAs that increase the radiosensitivity of esophageal cancer cells have shown that miR-34a reverses drug resistance through the SIRT1/PI3K/Akt/mTOR pathway [147], miR-338-5p targets survivin [148], the lncRNA NORAD promotes HRR via pri-miR-199a1/ATR/CHK1 [149], MAGI2-AS3 downregulates HOXB7 via EZH2/H3K27me3 [150], GAS5 increases apoptosis through RECK/miR-21 [151], and CCAT2 promotes resistance via Akt/ERK/p70S6K1 [152]. However, the instability of ncRNAs after delivery and their off-target effects limit their translational advancement. No mature RNA therapeutics for RT sensitization have been reported in esophageal cancer patients to date.

In terms of drug-mediated regulation of esophageal cancer radiosensitivity, studies have suggested that trace elements play crucial roles in the effects of RT on esophageal cancer. Copper (Cu) interacts with GLS2, thereby downregulating lipoic acid synthetase and dihydrolipoamide S-succinyltransferase, which leads to a reduction in the activity of the α-ketoglutarate dehydrogenase complex and blockage of the tricarboxylic acid cycle, ultimately increasing RT sensitivity [153]. One study revealed that the EV-STS delivery system can interact with ABCA1, thereby inhibiting the stem cell properties and radiation resistance of ESCC cells, which results in significant improvements in safety and efficacy at lower doses [154]. The SCD1 inhibitor MF-438 induces ferroptosis and ICD [155]. Additionally, lobaplatin increases RT responses by inducing apoptosis and modulating the PI3K/Akt pathway [156]. Simvastatin increases the radiosensitivity of esophageal cancer cells by modulating apoptosis via the PTEN-PI3K/Akt pathway [157]. Moreover, LBP (lobaplatin) promotes DNA damage, apoptosis, and cellular senescence [156]. Furthermore, selenium nanoparticles induce apoptosis through the p53/IGFBP3 pathway [158], and mannose significantly increases the rate of tumor cell apoptosis [159]. TM (tunicamycin) induces endoplasmic reticulum stress, which increases apoptosis and significantly activates autophagic responses [160].

**Figure 5:**
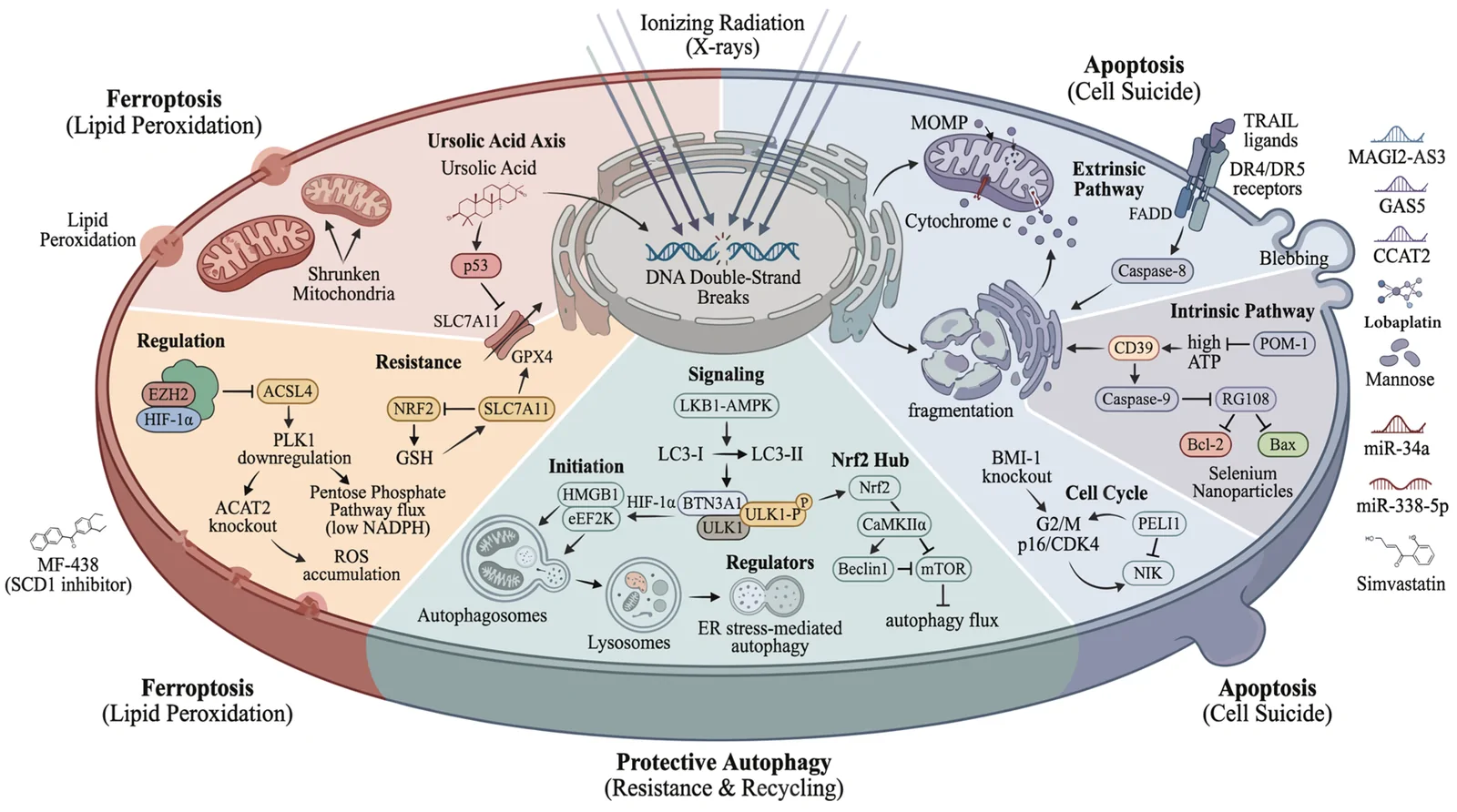
Partial mechanistic diagram of cell death and radiosensitivity in esophageal cancer cells (note: The pathways shown were compiled from independent studies and do not constitute a single experimentally validated signaling network.).

From a translational perspective, hierarchical evaluations have prioritized two classes of therapeutic candidates: first, clinically applicable agents, such as lobaplatin, whose synergistic radiosensitizing effects mediated by apoptosis have been validated in routine neoadjuvant regimens; and second, high-potential preclinical compounds targeting the shared Nrf2 hub (to simultaneously block autophagy and ferroptosis resistance) and TRAIL apoptotic pathways, which require further in vivo optimization before clinical translation. Despite these limitations, nearly all studies have focused on ESCC, with sparse mechanistic data describing cell death crosstalk in EAC, which is a key translational bottleneck. The related mechanistic diagrams are shown in Figure 5.

## 10. Biomarkers

RT is among the key treatments for esophageal cancer, especially in patients with locally advanced or inoperable tumors. However, there is significant individual variability in the responses of different patients to RT; therefore, the identification of effective biomarkers for predicting RT outcomes and assessing the efficacy of treatments is currently a research priority. Studies have gradually revealed several potential molecules and genes that are associated with the response to RT and patient prognosis.

As the most widely investigated category, predictive biomarkers identify pretreatment molecular and imaging features to stratify individual radiosensitivity and CRT responsiveness and include tissue molecular signatures, circulating noninvasive markers, and artificial intelligence-based radiomic models. At the tissue level, the ability of multiple ESCC-derived molecular markers to predict radiosensitivity has been validated. The quantification of tissue miR-21 expression by qRT–PCR in 63 paired ESCC samples revealed a correlation between tumor radioresistance and advanced TNM stage, with in vitro functional validation in multiple esophageal cancer cell lines under 6 Gy of irradiation; however, these findings lack external clinical validation, unified cutoff values, and reported sensitivity and specificity levels [161]. In a small cohort of 25 ESCC patients who received standardized preoperative RT, elevated pretreatment HDGF mRNA levels detected by semiquantitative RT–PCR corresponded to superior histopathological tumor regression, although validation was limited to internal cell and clinical datasets [162]. DNA damage repair molecules provide mechanistically robust predictive value: DNA-PK activity and Ku70 expression strongly correlate with intrinsic radiosensitivity in 14 esophageal cancer cell lines, and elevated nuclear DNA-PKcs expression detected by immunohistochemistry predicted a favorable CRT response in 67 retrospective ESCC patients [163,164]. FGF5 promoter hypermethylation is among the most well-validated tissue predictive biomarkers, achieving multi-cohort internal and external validation with an AUC of 71.2%, a sensitivity of 65%, and a specificity of 76% for pCR after definitive CRT [165]. Noninvasive circulating biomarkers offer superior clinical accessibility. The combination of the routine peripheral neutrophil count and serum TGF-β1 concentration was shown to predict the response to RT in 398 thoracic cancer patients, with an AUC of 0.84 and a C-index of 0.80 [166]. Additionally, serum miR-193b, serum miR-29b, and plasma exosomal miR-340-5b levels detected by qRT–PCR were found to serve as circulating predictive markers; however, all of these studies are restricted by their single-center retrospective design and insufficient external verification [83,167,168]. Liquid-derived methylation signatures also serve as predictive markers for treatment sensitivity stratification. Overall, radiomic predictive features (CT/PET/MRI-derived texture, shape, and intensity) achieved high pooled sensitivity and specificity (0.83–0.89) but relied heavily on internal validation with poor reproducibility [169]. Traditional radiomic features yield favorable pooled diagnostic performance but exhibit poor reproducibility and limited external validation.

Prognostic biomarkers focus on stratifying long-term survival outcomes and postoperative recurrence risk, with several circulating signatures exhibiting both predictive and prognostic roles in the clinical management of esophageal cancer. Most current prognostic biomarkers are minimally invasive blood-based indicators that reflect baseline tumor biological aggressiveness and treatment resistance. In patients with resectable EAC receiving standard CROSS-regimen neoadjuvant chemoradiation, an elevated pretreatment level of circulating TGF-β1 is associated with shortened progression-free survival and increased posttreatment metastatic progression and serves as a powerful prognostic indicator for metastatic risk stratification [170]. In another study, a tumor tissue-derived 9-gene classifier (APC, MAP3K6, ETS1, CSF3R, PDGFRB, GATA2, ARID1A, PML, and FGF6) effectively predicted relapse-free survival and overall survival in 34 patients with locally advanced EAC, with an AUC of 0.868, a sensitivity of 73.3%, and a specificity of 94.7%. However, the clinical utility of this panel is limited by the retrospective single-center enrollment and other inadequacies.

However, the clinical utility of this panel is constrained by its retrospective, single-center design [171]. In retrospective esophageal cancer cohorts, serum miR-193b and miR-29b independently predicted overall survival in advanced, surgery-ineligible patients who were undergoing definitive RT, providing stable prognostic stratification through standardized qRT–PCR detection [83,167]. Large-scale liquid biopsy reviews validated circulating tumor cell counts and ctDNA burden as auxiliary prognostic markers for esophageal cancer [172]. Nevertheless, existing prognostic biomarkers for esophageal cancer share prominent limitations. Most prognostic signatures are derived from single-center retrospective datasets without rigorous multicenter external validation. Stage-dependent detection bias, variable tumor heterogeneity, and the absence of unified clinical cutoff values further undermine the reproducibility and generalizability of these methods. In addition, insufficient mechanistic exploration and rare multivariate adjustments for confounding factors restrict their reliable application in standardized postoperative survival and recurrence risk stratification.

Diagnostic biomarkers facilitate primarily preoperative tumor screening, histological subtype discrimination, and posttreatment auxiliary diagnosis of residual lesions, supplementing conventional endoscopic and imaging examinations in the clinical workflow for esophageal cancer. The methylation panel consisting of miR129-2me, miR124-3me, and ZNF569me represents a high-performance diagnostic signature, particularly for EAC. After validation in 85 FFPE tissues and 62 plasma samples via qMSP detection, this triple-methylation signature achieved 90% sensitivity and 100% specificity in tissue samples and 53% sensitivity and 87% specificity in plasma samples, enabling accurate identification of esophageal cancer before and after chemoradiotherapy [173]. Broad-spectrum liquid biopsy platforms, including those involving ctDNA, extracellular vesicles, circulating autoantibodies, and FTIR spectroscopy, support large-scale pancancer screening for esophageal cancer. Compared with invasive tissue biopsy, liquid diagnostic biomarkers possess unique advantages, such as minimal invasiveness, convenient sampling, and repeated detectability, and are suitable for long-term disease monitoring. However, multiple challenges hinder their clinical translation. Current liquid biopsy biomarkers exhibit poor sensitivity for early-stage esophageal cancer, ambiguous tumor origin discrimination, inconsistent analytical performance across different platforms, and relatively high rates of false-positive and false-negative results. The lack of unified detection standards and prospective large-cohort validation further limits their stability and reliability in real-world diagnostic scenarios of esophageal cancer.

Treatment response biomarkers enable dynamic real-time monitoring of therapeutic efficacy and precise discrimination between residual viable tumors and pCR after neoadjuvant or definitive CRT, guiding individualized treatment adjustment and follow-up strategies. The triple-methylation signature (miR129-2me/miR124-3me/ZNF569me) reliably differentiates residual disease from complete response after neoadjuvant chemoradiation, providing molecular evidence for evaluating curative efficacy [173]. Plasma exosomal miR-340-5p is closely correlated with posttreatment in-field recurrence and acts as a dynamic surrogate biomarker for a sustained RT response [168]. Imaging-based biomarkers also exhibit excellent potential for evaluating treatment response. Compared with traditional PET/CT and endoscopy, DCE-MRI qualitative grading achieves superior diagnostic accuracy, with 73.5% sensitivity and 88.9% specificity for identifying pCR after neoadjuvant CRT and favorable interreader consistency [174]. Despite diverse available candidates, current treatment response biomarkers for esophageal cancer face universal translational obstacles. Most studies rely on retrospective single-center data without unified imaging acquisition or molecular detection standards. Insufficient prospective multi-institutional validation, unstable detection reproducibility, and inadequate biological interpretation severely restrict their clinical popularization, highlighting the urgent need for standardized research frameworks to bridge the gap between preliminary biomarker discovery and clinical implementation.

Therefore, future research should integrate basic and clinical studies, validate the clinical application values of these biomarkers, and improve the early detection and diagnosis processes employed for esophageal cancer patients. Priority should be given to advancing the clinical verification of low-cost, easily detectable circulating composite indicators and tissue DNA damage repair markers while unifying imaging acquisition standards to address the above technical and clinical limitations.

## 11. Limitations of the Study

This review systematically outlined the molecular mechanisms governing radiosensitivity in esophageal cancer under the radiobiological 6R framework; however, several limitations remain to be addressed. Literature retrieval was limited to the PubMed database and lacked standardized evidence grading, potentially reducing the overall comprehensiveness of the included studies. Owing to the scarcity of research focused on EAC, most regulatory signaling pathways summarized herein have been validated only in preclinical models of ESCC. As such, the translational extrapolation of these findings to EAC-predominant patient cohorts is limited. Given the primary focus of this manuscript on mechanistic profiling, layered critical appraisal of clinical data is relatively limited throughout the text. Biomarker-related evidence remains fragmented and lacks unified clinical cutoff values; liquid biopsy and radiomic models also face the technical bottlenecks of low tumor signal abundance and poor cross-center generalizability.

## 12. Conclusions

This review systematically elucidates the mechanistic landscape of esophageal cancer radioresistance on the basis of the classical 6R radiobiological principle. Multiple signaling cascades and epigenetic modulators collaboratively govern RT sensitivity, yielding numerous preclinical radiosensitizing targets and therapeutic strategies. Currently available evidence is heavily biased toward ESCC, with inadequate mechanistic data for EAC subtypes. Moreover, the clinical applicability of current molecular targets and predictive biomarkers requires further validation. Future translational studies are essential to elucidate subtype-specific resistance mechanisms and refine personalized RT regimens for esophageal cancer patients.

## Data Availability

No new data were created or analyzed in this study. Data sharing is not applicable to this article.
